# Family history of diabetes and its relationship with insulin secretion and insulin sensitivity in Iraqi immigrants and native Swedes: a population-based cohort study

**DOI:** 10.1007/s00592-017-1088-5

**Published:** 2017-12-22

**Authors:** Louise Bennet, Paul W. Franks, Bengt Zöller, Leif Groop

**Affiliations:** 1 Department of Clinical Sciences, Lund University, Skåne University Hospital, Malmö, Sweden; 2 Department of Family Medicine, Lund University, Skåne University Hospital, Malmö, Sweden; 3 Department of Diabetes and Endocrinology/Lund University Diabetes Centre, Skåne University Hospital, Malmö, Sweden; 4 Genetic and Molecular Epidemiology Unit, Lund University, Malmö, Sweden; 5 Department of Nutrition, Harvard School of Public Health, Boston, MA USA; 6 Department of Public Health and Clinical Medicine, Umeå University, Umeå, Sweden; 7 Center for Primary Health Care Research, Clinical Research Center, 28-11-015, Skåne University Hospital, Jan Waldenströms gata 35, 205 02 Malmö, Sweden

**Keywords:** Family history of diabetes, Insulin action, Insulin secretion, Middle East, Type 2 diabetes

## Abstract

**Aims:**

Middle Eastern immigrants to western countries are at high risk of developing type 2 diabetes. However, the heritability and impact of first-degree family history (FH) of type 2 diabetes on insulin secretion and action have not been adequately described.

**Methods:**

Citizens of Malmö, Sweden, aged 30–75 years born in Iraq or Sweden were invited to participate in this population-based study. Insulin secretion (corrected insulin response and oral disposition index) and action (insulin sensitivity index) were assessed by oral glucose tolerance tests.

**Results:**

In total, 45.7% of Iraqis (616/1348) and 27.4% of native Swedes (201/733) had FH in parent(s), sibling(s) or single parent and sibling, i.e., FH+. Approximately 8% of Iraqis and 0.7% of Swedes had ≥ 3 sibling(s) and parent(s) with diabetes, i.e., FH++. Irrespective of family size, prediabetes and diabetes increased with family burden (FH− 29.4%; FH+ 38.8%; FH++ 61.7%) without significant differences across ethnicities. With increasing level of family burden, insulin secretion rather than insulin action decreased. Individuals with a combination of ≥ 3 siblings and parents with diabetes presented with the lowest levels of insulin secretion.

**Conclusions:**

The Iraqi immigrant population often present with a strong familial burden of type 2 diabetes with the worst glycemic control and highest diabetes risk in individuals with ≥ 3 siblings and parents with diabetes. Our data show that in a population still free from diabetes familial burden influences insulin secretion to a higher degree than insulin action and may be a logical target for intervention.

## Introduction

Throughout the past few decades, political instability, persecution and war in some regions of the Middle East have caused millions of people to flee their homelands and seek refuge in Northern Europe. Of all countries worldwide, Sweden is one of the biggest per capita recipients of refugees. The city of Malmö is currently home to one of the largest Middle Eastern communities in Sweden, with a third of the city’s citizens having been born outside Sweden, most in Iraq [1]. The next decade is likely to witness a substantial increase in the number of refugees and economic migrants from Iraq, Afghanistan and Syria entering Sweden. Thus, there is a growing need to understand the health characteristics of these populations, identify the major risk factors for cardiometabolic disease and establish effective primary and secondary prevention strategies.

People who migrate to western societies often develop cardiometabolic diseases more rapidly than endogenous populations, predominantly owing to socioeconomic vulnerability, the uptake of unhealthy lifestyle and genetic susceptibility [2, 3].

Family history of diabetes is a proxy for genetic risk [4]. In people of European ancestry, type 2 diabetes risk increases with number and type of family members with diabetes, with the highest relative risk of type 2 diabetes in individuals with at least two affected siblings [5]. Further, first-degree family history of diabetes is associated with impaired insulin secretion and action [6]. In the MEDIM (the impact of Migration and Ethnicity on Diabetes In Malmö) population-based study conducted in Malmö, Sweden, we have previously reported that Middle Eastern immigrants from Iraq represent a high-risk population for type 2 diabetes and that diabetes-related risk factors such as family history of diabetes, obesity and insulin resistance cluster in this population [7, 8]. Further, the MEDIM has shown that family history and obesity are strongly associated with earlier diabetes onset in Middle Eastern immigrants [7]. However, the number and type of affected family members with diabetes have not been adequately described and compared in a population of Middle Eastern versus a population of European ancestry. Further, the impact of familial risk on insulin secretion, insulin action and type 2 diabetes has not been studied and compared across populations of Middle Eastern and European ancestries, which is the aim of this study to investigate.

## Methods

### Aims

In this cohort comprising people born in Iraq or Sweden, our aim was to study number and type of family members (parents and/or siblings) with type 2 diabetes. A further aim was to investigate the associations of familial risk of type 2 diabetes with insulin sensitivity and beta-cell function and compare these associations between Iraqi immigrants and native Swedes.

### Characteristics of participants and sampling process

The design and sampling has been described previously [8]. Briefly, citizens of Malmö born in Iraq or Sweden aged 30–75 years were randomly selected from the census register and invited by mail and phone to participate in this population-based survey. We aimed to recruit Swedish participants matched for sex and age distributions living in the same geographical area in Malmö. People with missing data on first-degree family history of diabetes or with severe physical or mental illness or other disabilities that would prevent them from fully engaging in the study were excluded.

**Fig. 1:**
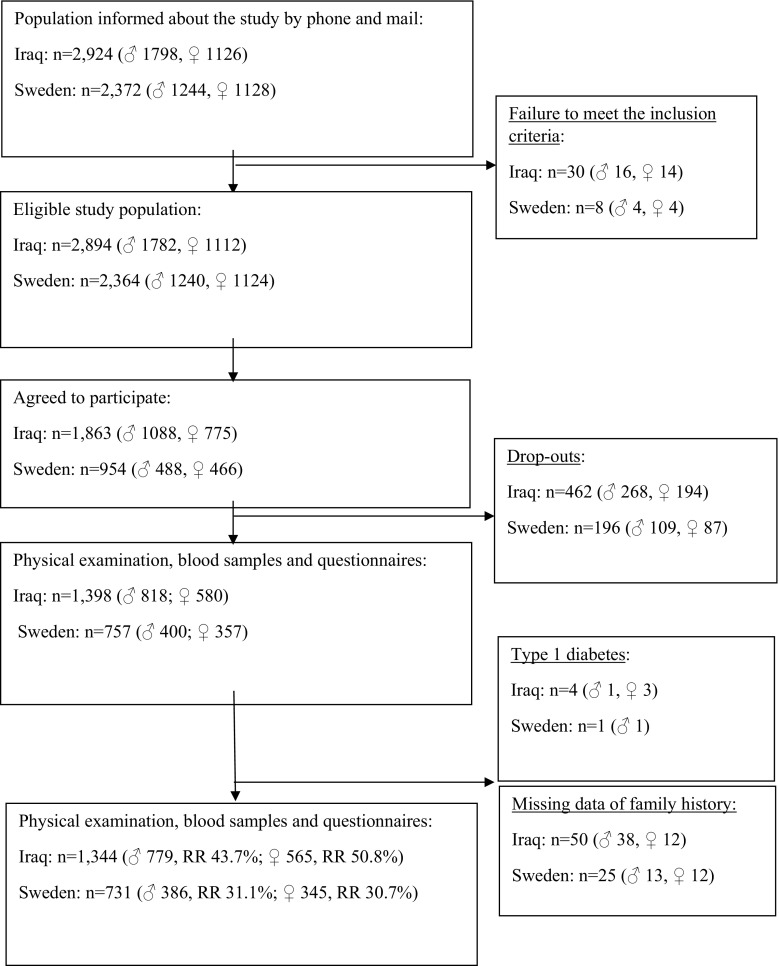
Flow chart describing the recruitment of MEDIM participants and response rate

Environmental conditions can influence population demography, i.e., ‘cohort effects’ [9]. To minimize cohort effects and assessment biases, examinations were conducted within a relatively short timeframe (February 1, 2010, through December 31, 2012). A flow chart describing the recruitment of MEDIM participants and response rate is described in Fig. 1.

### Measures

Standard physical examinations, including clinical variables such as height, weight, waist circumferences, BMI, fasting blood samples, oral glucose tolerance test (OGTT), insulin sensitivity index (ISI), insulin secretion (corrected insulin response, CIR) were assessed as previously described [8]. Questionnaires gathered information on comorbidity, medication, socio-economy and lifestyle as described previously [8].

*First-degree family history of diabetes*: This information was self-reported and gathered through questionnairs. Family history was considered as the presence of diabetes in biological parents and/or siblings and/or children. Previous studies have shown that familial relative risk of diabetes depends on the number and type of affected family members with diabetes [5]. Accordingly, in this study familial relative risk was considered in relation to the odds of type 2 diabetes (Table 3). The degree of familial risk was categorized as follows: FH− (no first-degree relatives with diabetes); odds of type 2 diabetes < 4.0, FH+ (family history of diabetes in parent(s), sibling(s) or single parent and sibling); odds of type 2 diabetes > 4.0, FH++ (family history of diabetes in a combination of ≥ 3 sibling(s) and parent(s) with diabetes). Since information is lacking in this study about whether relatives have type 1 diabetes or type 2 diabetes, we simply refer to ‘family history of diabetes’ of which the majority is estimated to have type 2 diabetes [10].

*Prediabetes* Participants with impaired fasting glucose (IFG), impaired glucose tolerance (IGT) or type 2 diabetes (T2D). IFG was defined as fasting plasma glucose (f-glc) > 6.1 mmol/L but < 7.0 mmol/l and a 2-h glc < 7.8 mmol/L. IGT was defined as f-glc < 6.1 mmol/L and a 2-h glucose ≥ 7.8 mmol/L but < 11.1 mmol/L. Impaired glucose regulation (IGR) was considered in those with a combination of IFG and IGT.

*Type 2 diabetes* Participants with previously known diabetes confirmed by medication with oral hypoglycemic agents and/or insulin or by a fasting plasma glucose value of ≥ 7.0 mmol/L were considered as having diabetes and did not undergo an OGTT. New cases of type 2 diabetes were confirmed by a fasting plasma glucose value of ≥ 7.0 mmol/L and/or by a 2-h plasma glucose value of ≥ 11.1 mmol/L [11]. If only one glucose value was pathologic, the OGTT was repeated on another day within 2 weeks with the same fasting procedures. Two values exceeding these thresholds were needed for diagnosis [11]. Diabetes cases with onset before the age of 20 years and/or antibodies against glutamic acid decarboxylase (GAD) were considered as type 1 diabetes/latent autoimmune diabetes (LADA) in the adult and were excluded from the study (Fig. 1).

#### *Hyperglycemia*: participants with prediabetes or type 2 diabetes

Insulin action and secretion: ISI, CIR and oral disposition index (DIo) were derived from the OGTT and assessed using the Matsuda indices [12]. *Insulin sensitivity index (ISI)*, *corrected insulin response* (*CIR*) and *oral disposition index* (*DIo*) were assessed using the Matsuda indices that were calculated from the OGTT results as follows:

ISI = 10,000/√ [(f-glc (mmol/L) × f-insulin (mIE/L)) × (mean OGTT glc conc. (mmol/L) × mean OGTT insulin conc. (mIE/L))] [12].

CIR is a measure of glucose-stimulated insulin secretion at 30 min of OGTT and provides an estimation of beta-cell function and was calculated as follows:

CIR = (100 × insulin at 30 min (mIE/L)/(glc30 (mmol/L) × (glc30 - 3.89 mmol/L)) [13] and requiring that glucose at 30 min (glc30) > 4.44 mmol/l and glc30 > f-glc [14].

DIo provides an estimate of beta-cell function adjusted for insulin resistance and takes the degree of insulin sensitivity into account as CIR is driven by both glucose and insulin sensitivity. DIo is calculated as CIR multiplied by ISI [15].

*Physical activity* was self-reported and quantified in hours per week as previously reported [8].

*Soda consumption* was self-reported. Regular intake was considered for those reporting intake almost every day during the week.

*Iraqi food preference* was self-reported and considered in those reporting that they eating food cooked according to recipes used in Iraqi cooking. This food is reported contain a high percentage fat [16].

### Statistical analyses

Analyses were performed using Stata IC/12.1. Skewed variables were log_10_-transformed before analysis to approximate normal distributions. Differences in means of anthropometric measures, fasting blood samples, insulin sensitivity and insulin secretion between categories of family history were examined by linear regression analysis adjusting for age and sex, whereas differences between proportions were assessed using logistic regression, presenting *P* for trend (Table 1). The odds ratios (OR) of type 2 diabetes were assessed using logistic regression, data were presented as OR 95% CI (Table 3) and *P*-values between categories were calculated from the logistic regression equation. To adjust for multiple testing, Bonferroni post hoc correction was assessed with corrected *P* = 0.0065 (0.05/8 tests) considered significant (Table 3).

**Table 1:** Characteristics of the study population in relation to the absence (FH−) or presence of family history of diabetes in mother, children, siblings single sibling and single parent (FH+) or diabetes in both parents and siblings, parents and children, single parent and siblings (FH++)

Variable	Born in Iraq				Born in Sweden			
	FH−	FH+	FH++	*P* for trend	FH−	FH+	FH++	*P* for trend
	*N* = 623	*N* = 616	*N* = 109		*N* = 526	*N* = 201	*N* = 6	
Age (years)	45.4 (9.5)	46.5 (9.6)	49.2 (9.2)	< 0.001	49.4 (11.5)	49.9 (10.6)	55.6 (9.1)	0.328
Male sex, *n* (%)	374 (60)	348 (56.5)	58 (53.2)	0.106	288 (54.8)	97 (48.3)	3 (50)	0.126
Waist men (cm)	98.7 (11.2)	99.9 (10.1)	99.9 (9.5)	0.149	97.7 (12.0)	97.8 (10.5)	93.7 (4.6)	0.892
Waist women (cm)	91.5 (10.6)	94.1 (11.1)	96.4 (11.8)	0.001	87.2 (13.4)	97.7 (12.1)	94.7 (10.0)	< 0.001
Body mass index (kg/m^2^)	28.9 (4.4)	29.5 (4.7)	29.9 (4.5)	0.002	26.9 (4.6)	27.8 (4.9)	28.4 (4.3)	0.018
F-glucose (mmol/L)	5.7 (1.2)	6.0 (1.5)	6.9 (2.6)	< 0.001	5.7 (1.2)	5.9 (1.2)	6.2 (0.9)	0.042
2-h glc (mmol/L)	5.8 (2.2)	6.3 (2.5)	6.5 (2.2)	< 0.001	5.8 (2.3)	6.2 (2.9)	8.1 (1.5)	0.013
F-insulin (mmol/l)	11.2 (7.2)	12.1 (7.9)	13.9 (12.9)	0.001	9.4 (8.1)	9.6 (5.7)	10.0 (4.0)	0.652
2-h insulin (mmol/l)	53.9 (52.4)	62.3 (58.3)	65.5 (69.9)	0.008	44.9 (59.7)	44.0 (37.3)	70.0 (44.6)	0.894
C-peptide (mmol/L)	0.80 (0.32)	0.84 (0.32)	0.87 (0.42)	0.013	0.71 (0.34)	0.76 (0.33)	0.81 (0.16)	0.060
HbA1c (mmol/mol)	36.6 (7.9)	38.4 (10.7)	42.8 (14.0)	< 0.001	35.9 (8.1)	37.2 (8.3)	37.1 (3.6)	0.080
PA, h/week^a^	2.0 (2.1)	1.8 (2.1)	2.4 (2.1)	0.555	4.0 (2.5)	4.0 (2.4)	2.2 (2.0)	0.489
Regular soda intake, n(%)	370 (59.4)	368 (59.7)	58 (53.2)	0.778	154 (29.3)	65 (32.3)	2 (33.3)	0.219
Iraqi food preference	596 (95.7)	582 (94.5)	105 (96.3)	0.648	–	–	–	–
ISI (mmol/L*mIE/L^−1^)^a^	77.7 (52.6 – 118.7)	72.7 (47.4 – 108.9)	78.5 (48.5 – 114.5)	0.063	106.4 (72.7 – 161.4)	87.2 (56.3 – 144.4)	53.2 (49.2 – 116.3)	0.004
CIR (mmol/L*mmol/L*mIE/L – 1^a,b^	177.8 (102.7–305.0)	167.2 (92.3–275.2)	129.4 (75.3–209.2)	0.033	146.0 (85.4–237.2)	128.7 (68.9–215.2)	89.1 (34.4–200.6)	0.023
DIo(mmol/L*mmol/L*mmol/L)^a,b^	13525.1 (7355.7–24699.8)	11973.4 (6249.2 – 22397.5)	9403.5 (5019.5–14101.8)	0.002	15106.2 (8867.2 – 26021.6)	10627.9 (6147.4 – 22449.7)	8321.5 (2619.4 – 12199.5)	< 0.001
IFG, IGT or T2D	185 (29.7)	241 (39.1)	66 (60.6)	< 0.001	153 (29.1)	76 (37.8)	5 (83.3)	0.004
T2D	47 (7.5)	73 (11.9)	36 (33.0)	< 0.001	26 (4.9)	17 (8.5)	1 (16.7)	0.031

Data presented in means (standard deviation, SD), numbers (percentages) or medians (interquartile range, IQR)

*CIR* corrected insulin response; *DIo* disposition index; *HDL* high-density lipoprotein; *ISI* insulin sensitivity index

^a^Log_10_-transformed and data presented as medians with IQR

^b^CIR and DI only included cases where the glucose level at 30 min was > 4.44 mmol/l and greater than the fasting glucose level [14]

Associations with ISI and DIo (base 10 log-transformed) were estimated using multivariable linear regression analysis; data are expressed as beta-coefficients (*β*) with 95% CI’s (Table 4). Regression coefficients were standardized to a unit variance (S.D.) in the strata of ethnicity and sex for the independent variables using z-score transformation (with a mean of 0 and standard deviation (SD) of 1). To determine whether independent variables included in the linear regression modified the primary associations of interest, tests for interactions were performed. Multicollinearity was tested for but was not considered an issue as all variance inflation factors (VIP) in the multivariate regression models had values < 1.6 [17].

All tests were two-sided, and a *p* value of < 0.05 was considered statistically significant.

## Results

### Family history of type 2 diabetes in Iraqi- and Swedish-born participants

In total, 1348 eligible participants born in Iraq and 733 born in Sweden participated and answered the question regarding first-degree family history. Iraqi immigrants had larger families in general with participants reporting having a median of six siblings (0–17) and three children (0–13); the corresponding numbers in Swedes were two siblings (0–9) and one child (0–5).

Distribution of family history in parents and siblings is presented in Table 2. The odds of type 2 diabetes in relation to first-degree family history of diabetes is presented in Table 3. A considerably larger proportion of Iraqi- than Swedish-born participants had a history of diabetes in parent(s), sibling(s) or single parent and sibling (45.7 vs. 27.4%, *P* < 0.001) (Table 2). These participants were defined as FH+ (Table 3). Further, ten times more Iraqis than native Swedes had a history of diabetes in a combination of ≥ 3 sibling(s) and parent(s) (7.7 vs. 0.7%, *P* < 0.001), Table 2. These participants were defined as FH++ (Table 3). In this study, 94.8% (109/115) of FH++ participants are represented by Iraqi immigrants. Due to few cases (< 1%), participants with children with diabetes were not considered in the further analysis.

**Table 2:** Population-based prevalence (%) of first-degree family history (FH) of diabetes in immigrants from Iraq versus native Swedes

Family member with diabetes	Total study population	
	Born in Iraq	Born in Sweden
	*N* = 1348	*N* = 733
No first-degree FH, % (*N*)	46.2 (623)	71.8 (526)
First-degree family history, % (*N*):		
Father	10.2 (138)	10.0 (73)
Mother	13.3 (179)	9.5 (70)
Both parents	4.4 (59)	1.1 (8)
Single sibling	5.8 (78)	4.2 (31)
Single sibling and single parent	8.3 (112)	1.5 (11)
Two or more siblings	3.1 (41)	0.1 (1)
Two or more siblings and single parent	3.9 (53)	0.4 (3)
One or more siblings and both parent	3.8 (52)	0.3 (2)
Child(ren)	0.7 (9)	1.0 (7)
Child(ren) and parent(s)	0.3 (4)	0.1 (1)

### Family history and phenotype by ethnicity

In both Iraqis and native Swedes, the prevalence of prediabetes and type 2 diabetes increased with increasing family burden (Table 1). In Iraqi-born participants fasting glucose, 2-h glucose, fasting insulin, 2-h insulin, C-peptide and HbA1c increased with increasing number of family members with diabetes. This trend was also observed for the Swedish-born participants, however, not statistically significant. Iraqis had higher soda intake and lower physical activity levels than those born in Sweden, even in individuals with no family history of diabetes. Lifestyle including self-reported food preference, soda consumption and physical activity did not appear to differ according to degree of family history in either cohort.

In both cohorts, insulin secretion response (assessed as CIR and DIo) was lower in FH+ than FH− participants with the lowest insulin secretion in FH++ participants (Table 1). In general, insulin action was lower in Iraqis than in Swedes (76 vs. 100.8 mmol/L mIE/L^−1^, *P* < 0.001, age, sex and family history adjusted data). With increasing family burden, insulin action decreased only in the native Swedish population but not in the Iraqi-born group (Table 1).

### Type 2 diabetes, insulin secretion and insulin action in relation to familial burden

**Fig. 2:**
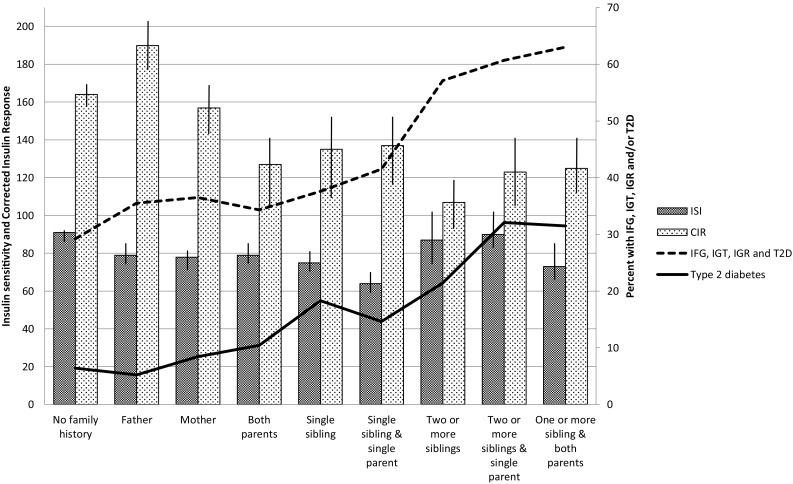
Prediabetes and type 2 diabetes in relation to insulin secretion and insulin action and degree of family history of diabetes. Vertical lines indicate 95% confidence intervals

In the total study population, insulin secretion was highest in those with a single father with diabetes, lower in those with a mother with diabetes and lower still in those with both parents affected. In participants with affected parent(s) and sibling(s), the insulin secretion was further decreased. Insulin action did not change considerably with familial burden (Fig. 2).

The prevalence of hyperglycemia increased with number of first-degree family members with diabetes and exceeded 30% in FH+ participants and exceeded 50% in FH++ participants (Fig. 2). The prevalence of type 2 diabetes in FH+ participants varied between 5 and 20% and in FH++ participants exceeded 30% (Fig. 2).

FH + participants had approximately twice the odds of type 2 diabetes, whereas FH++ participants had more than four times the odds of type 2 diabetes, compared to FH− participants, adjusted for multiple testing (Table 3). The highest odds ratios (> 4.0) were observed in participants with both parents and at least one siblings affected by diabetes. There were no detectable interactions between country of birth and family history.

**Table 3:** Odds ratios for type 2 diabetes in relation to first-degree family history of diabetes assessed by logistic regression estimating odds ratios (ORs) and 95% confidence intervals (CIs)

Odds ratios of type 2 diabetes		
Covariates	OR	95% CI
No family history of diabetes	Reference	
First-degree family history^*^:		
FH+		
Father	0.88	0.45–1.74
Mother	1.44	0.87–2.54
Both parents	1.90	0.79–4.54
Single sibling	**1.84**	1.01–3.36
Single sibling and single parent	**1.83**	1.01–3.34
Two or more siblings	2.13	0.92–4.92
FH++		
Two or more siblings and single parent	**4.62** ^*^	2.32–9.18
One or more sibling and both parents	**5.74** ^*^	2.89–11.40
Age, per 1 SD	**2.20**	1.86–2.60
Male Sex	**1.63**	1.16–2.29
BMI, per 1 SD	**1.76**	1.51–2.04
Born in Iraq	**1.89**	1.15–3.11
Number of siblings	1.01	0.92–1.07

Participants with family history in children omitted due to few cases

Units were standardized in the strata of ethnicity and sex per 1 standard deviation (SD) unit variance for the continuous independent variables

*Bonferroni post hoc correction *P* < 0.006 (0.05/8 tests)

Significant OR is bolded

### Family history, insulin action and insulin secretion

In the total study population, there was a positive relationship between insulin secretion (assessed as DIo) and family history of diabetes, with higher beta coefficients for FH++ compared with FH+ individuals (Table 4). The associations remained statistically significant after adjusting for the putative confounding effects of age, sex, waist circumference, country of birth and lifestyle (physical activity).

**Table 4:** Association between family history of diabetes (FH) with insulin secretion (DIo) and insulin sensitivity (ISI) in the total study population of Iraqi and Swedish born participants

Disposition index^a^							
	FH−	FH+			FH++		
		*β*	95% CI		*β*	95% CI	
Model I	Ref.	**−** .**077** ^***^	**−** .117	**−** .037	**−** .**160** ^***^	**−** .259	**−** .062
Model II	Ref.	**−** .**060** ^**^	**−** .100	-.021	**−** .**142** ^**^	**−** .238	**−** .047
Model III	Ref.	**−** .**058** ^**^	**−** .100	-.017	**−** .**172** ^***^	**−** .271	**−** .073

Insulin sensitivity index^a^							
	FH-	FH+			FH++		
		*β*	95% CI		*β*	95% CI	
Model I	Ref.	**−** .**042** ^**^	**−** .068	-.016	**−** .044	**−** .107	.019
Model II	Ref.	**−** .021	**−** .043	.002	**−** .020	**−** .074	.034
Model III	Ref.	**−** .022	**−** .045	.002	**−** .024	**−** .079	.032

Data assessed by multivariate linear regression displaying *β* coefficients with 95% confidence intervals (CI) for FH+ and FH++ participants with FH− as reference (Ref.) and ISI and DIo as dependent variables

FH− No family history of diabetes

FH+ History of diabetes in parent(s), sibling(s) or single parent and sibling

FH++ History of diabetes in a combination of ≥ 3 sibling(s) and parent(s)

Model I to III

Model I Age, male gender, born in Iraq

Model II Age, male gender, born in Iraq, waist circumference

Model III Age, male gender, born in Iraq, waist circumference, physical activity

Units: age (years); physical activity (hours/week physically active), ISI (mmol/L mIE/L)^−1^

Units were standardized in the strata of ethnicity and sex per 1 standard deviation (SD) unit variance for the continuous independent variables

^a^Base 10 log-transformed

^*^ *p* < 0.05, ^**^ *p* < 0.01, ^***^ *p* < 0.001

The analyses included participants completing an OGTT and cases where glucose was measured at 30 min (glc30) > 4.44 mmol/L and glc30 > fasting glucose [14]

Participants with missing data for any of the included variables were excluded from the analysis

The associations between insulin action assessed as ISI and degree of family history were not statistically significant for FH+ and FH++ participants. The association between insulin action and FH+ was no longer significant after adjusting for waist circumference. There were no significant interactions between family history and country of birth.

## Discussion

### Key findings

To the best of our knowledge, this is the first study to investigate the impact of number and type of diabetes affected family members on insulin secretion and action, in a Middle Eastern immigrant versus a native Swedish population. The Iraqi immigrant population often present with a strong familial burden of type 2 diabetes. Irrespective of family size, individuals with a combination of ≥ 3 siblings and parents with diabetes present the worst glycemic control and highest diabetes risk. Another key finding is that insulin secretion rather than insulin action decreases with number and type of family member affected with the lowest levels of insulin secretion in individuals with combination of ≥ 3 siblings and parents with diabetes. Dysfunctional insulin secretion is a key factor to diabetes development in insulin-resistant individuals [18], and a plausible explanation to the very high prevalence of prediabetes and type 2 diabetes in individuals with ≥ 3 sibling(s) and parent(s) with diabetes, that to 95% is represented by Iraqi immigrants.

### Family history, type 2 diabetes and insulin secretion

Family history, obesity, impaired insulin action and secretion are strong predictors of type 2 diabetes [4, 6]. These are risk factors that, as we have previously reported, cluster in the Iraqi immigrant population in Sweden [8, 19]. The incapacity of beta-cells to compensate for the impaired insulin action is previously identified as the key defect leading to subsequent type 2 diabetes [4]. A previous studies conducted in Finland have reported reduced insulin secretion and increased type 2 diabetes risk among those with a family history of diabetes [20]. Family history comprises a variety of factors that are associated with diabetes through shared environmental factors (such as education, socioeconomic situation, lifestyle habits, obesity) or genetic factors [21]. A positive family history is shown to correlate with a genetic risk of type 2 diabetes (carriers of > 12 alleles for type 2 diabetes) [6]. Recent studies have further shown that genetic risk variants for type 2 diabetes are more strongly associated with defect insulin secretion rather than insulin action [6, 22]. Altogether, our findings of a high familial burden in Iraqi immigrants, the strong influence of family history on insulin secretion rather than on insulin action, together with the higher relative diabetes risk, could indicate a higher burden of known or unknown genetic variants influencing insulin secretion in the Iraqi immigrant population, but remains to be studied further.

One could argue that the high prevalence of family history in the Iraqi-born population is a consequence of larger families with more siblings and children, but the data studying associations between family history and diabetes risk are adjusted for number of siblings and our data are thus not likely to be biased by family size. Further, irrespective of number of children and siblings, the majority of Iraqis had one or both parents affected by diabetes (44.2% of Iraqis and 22.9% of Swedes), indicating that the familial burden is truly higher in the Iraqi than native Swedish population.

### Family history and insulin action

The association between family history and type 2 diabetes is previously reported to be predominantly explained by shared environmental and genetic components influencing behavior, lifestyle and metabolism [21]. Although study size impacts the extent to which some of the hypothesis of interest here can be tested, this study reports an indirect rather than direct effect of family history on insulin action, with waist circumference explaining a large proportion of the variance between family history and insulin action which is in agreement of data reported previously [20].

Previous studies have reported that the genetic predisposition to type 2 diabetes is mediated by its effect on obesity [23]. In populations of different ancestry such as African Asian, South Asian, Caucasian and Pima Genetic, predisposition to, for instance, the *FTO* gene is confirmed to predispose for obesity [24]. However, genetic epidemiology using Mendelian randomization has also shown an obesity-dependent association between the *FTO* gene and type 2 diabetes [25] and that the *FTO* gene predisposes to insulin resistance [26]. In the Swedish population, insulin action seemed to be lower in those with a high familial burden. However, only a few percent of native Swedes had a high familial burden of type 2 diabetes and our data were not powered to detect significant differences across ethnicities in the association between familial burden and insulin action. Future studies should focus on unraveling epigenetic mechanisms and uncovering causal factors underlying type 2 diabetes in this Middle Eastern population representing one of the largest growing immigrant groups in Sweden and Europe of today.

### Strengths and limitations

Over twenty percent of the total Iraqi immigrant population in this age group in the city of Malmö participated in this population-based study with no difference in prevalence of type 2 diabetes between participants and non-participants indicating a high representativeness of the study population and low risk of selection bias [8]. The study includes detailed metabolic phenotyping, lifestyle and family history assessments with insulin secretion and action assessed from OGTT and Matsuda indices. One could argue that self-reported data on first-degree family history could be a limitation; however, self-reported data have shown to be more accurate in this population as compared to the native Swedish population [27]. Due to the fact the participants have migrated from Iraq, it was not possible to collect register data on their relatives; however, the consistent results in this study with previous studies of the prevalence of family history of diabetes and familial risk of type 2 diabetes [5, 21, 28] confirm the high validity of our assessments.

A potential weakness is the cross-sectional design making it difficult to draw conclusions regarding causality. The samples size may influence the outcome of our data with insignificant interactions across ethnicity and family history. The low number of Swedes with less than 1% having both parents and siblings, single parent and siblings, parents and children affected by diabetes made it impossible to study the risk of family clustering on insulin secretion and action.

## Conclusions

This study concludes that Iraqi immigrants present with a strong familial burden for type 2 diabetes. Dysfunctional insulin secretion is a key factor to diabetes development in insulin-resistant individuals [18], and a plausible explanation to the very high prevalence of prediabetes and type 2 diabetes in individuals with a strong family history, that to 95% are represented by Iraqi immigrants. Determination of ethnic background and number of first-degree relatives with diabetes are easily captured in health care and should be considered in the preventive work against future type 2 diabetes in Middle Eastern populations.

## Abbreviation

CIR: Corrected insulin response
DIo: Oral disposition index
FH: Family history
ISI: Insulin sensitivity index
